# Recreational Screen Time at University Entry and Mental Health and Well-Being Over First Year: U-Flourish Student Well-Being Research: Temps d’écran à des fins de divertissement au moment de l’entrée à l’université, santé mentale et bien-être au cours de la première année : programme de recherche U-Flourish sur le bien-être des étudiants

**DOI:** 10.1177/07067437261428821

**Published:** 2026-03-06

**Authors:** Simran Brar, Nathan King, Anna Park, Kristen Kyone, Emily Dephoure, Daniel Rivera, Adeleine Lyon, Anne Duffy

**Affiliations:** 1 Department of Public Health Sciences, 5620 Queen's University, Kingston, ON, Canada; 2 Department of Psychology, McGill University, Montreal, QC, Canada; 3 Department of Psychiatry, Queen's University, Kingston, ON, Canada; 4 Faculty of Health Sciences, 4257 Queen's University, Kingston, ON, Canada; 5 School of Medicine, 4257 Queen's University, Kingston, ON, Canada; 6 Centre for Neuroscience Studies, Queen's University, Kingston, ON, Canada; 7 Department of Psychiatry, Oxford University, Oxford, UK

**Keywords:** screen time, well-being, mental health, university students, depression, anxiety, insomnia, eating disorders, heath promotion

## Abstract

**Background:**

Mental health concerns are common among undergraduates and have been linked to lifestyle factors. This study examined the relationship between recreational screen time at university entry and mental health over the academic year, and the potential moderating role of self-esteem.

**Methods:**

Data from the longitudinal Queen's U-Flourish Survey (2021/2022) included measures of screen time unrelated to academic work (leisure and social) and validated screening measures for anxiety (GAD-7), depression (PHQ-9), insomnia (SCI-8), disordered eating (SCOFF), and low well-being (SWEMWBS). Multivariable log-binomial regression was used to examine associations between screen time and mental health.

**Results:**

At entry to university, a higher proportion of males (n = 394) than females (n = 1,135) reported averaging 4 +  hours/day of leisure screen time (57.4% vs. 45.3%, *P* < 0.001), while a greater proportion of females reported 4 +  hours of social screen time (32.2% vs. 25.2%, *P* = 0.01). Higher screen time was associated with increased risk of clinically significant levels of symptoms and reduced well-being at school entry, with greater effects for leisure compared to social screen time. Students reporting 7–9 and 10 +  hours/day of leisure screen time were more likely to screen positive for anxiety (by 20% and 39%), depression (39%; 47%), insomnia (22%; 55%), and low well-being (45%; 68%) compared to students averaging ≤3 hours/day. Effects were comparable between males and females, except greater leisure screen time was associated with increased risk of disordered eating among females only. Associations between screen time and screening positive at the end of the academic year were largely attenuated. Leisure screen time ≥3 hours/day was most strongly associated with anxiety and depression among students with higher self-esteem.

**Conclusions:**

Recreational screen time ≥3 hours/day is common among first-year undergraduates and associated with higher levels of anxiety and depressive symptoms and lower well-being. It should be considered in campus mental health promotion and prevention efforts.

## Résumé

**Contexte:**

Les problèmes de santé mentale, courants chez les étudiants de premier cycle, ont été associés au mode de vie. Cette étude a porté sur le lien entre le temps passé devant un écran à des fins de divertissement au moment de l’entrée à l’université et la santé mentale au cours de l’année scolaire, ainsi que sur le rôle modérateur que peut potentiellement jouer l’estime de soi.

**Méthodes:**

Les données de l’enquête longitudinale U-Flourish menée à l’Université Queen’s (2021-2022) comprenaient des mesures du temps passé devant un écran sans rapport avec le travail scolaire (loisirs et réseaux sociaux), ainsi que des tests reconnus pour le dépistage de l’anxiété (GAD-7), de la dépression (PHQ-9), de l’insomnie (SCI-8), des troubles alimentaires (SCOFF) et des problèmes de bien-être mental (WEMWBS-7). La régression logistique binomiale multivariable a été utilisée pour étudier les liens entre le temps d’écran et la santé mentale.

**Résultats:**

À leur entrée à l’université, une plus grande proportion d’hommes (n = 394) que de femmes (n = 1 135) ont déclaré avoir passé en moyenne 4 heures ou plus par jour devant un écran à des fins de loisirs (57,4 % contre 45,3 %, *p* <* 0,001*), tandis qu’une plus grande proportion de femmes a déclaré avoir passé 4 heures ou plus devant un écran pour accéder aux réseaux sociaux (32,2 % contre 25,2 %, *p*=*0,01*). Plus de temps d’écran a été associé à un risque accru de symptômes cliniquement significatifs et à une réduction du bien-être au moment de l’entrée à l’école; par ailleurs, les effets étaient plus importants lorsque ce temps d’écran était employé à des fins de loisirs plutôt qu’aux réseaux sociaux. Les étudiants qui ont déclaré avoir passé entre 7 et 9 heures ou 10 heures et plus par jour devant un écran à des fins de loisirs étaient davantage susceptibles de présenter des résultats positifs aux tests de dépistage de l’anxiété (20 % et 39 %), de la dépression (39 %; 47 %), de l’insomnie (22 %; 55 %) et des problèmes de bien-être mental (45 %; 68 %) que ceux qui avaient passé en moyenne 3 heures ou moins par jour devant les écrans. Les effets étaient comparables entre les hommes et les femmes. La seule différence était qu’un temps d’écran prolongé à des fins de loisirs n’était associé à un risque accru de troubles de l’alimentation que chez les femmes. Les liens entre le temps d’écran et les résultats positifs aux tests de dépistage en fin d’année scolaire se sont en grande partie atténués. Un temps d’écran à des fins de loisirs de 3 heures ou plus par jour était le plus fortement associé à de l’anxiété et à une dépression chez les étudiants qui avaient une plus grande estime de soi.

**Conclusions:**

Les étudiants de premier cycle passent fréquemment 3 heures ou plus par jour devant un écran à des fins de divertissement, et cette tendance est associée à des niveaux plus élevés d’anxiété et de symptômes dépressifs et à un plus faible bien-être. Il faudrait en tenir compte dans les campagnes de promotion et de prévention en matière de santé mentale menées sur les campus.

## Introduction

The transition to university is often a challenging time for young people, as they take on greater responsibility for managing their time, health, finances, and relationships while meeting the demands of higher education.^1,2^ Mental health concerns are common among undergraduates, with approximately one-third to one-half screening positive for anxiety and depression.^3,4^ The COVID-19 pandemic, marked by social restrictions and campus closures, exacerbated these problems among Canadian university students.^4–7^ Mental health challenges are, in turn, associated with academic difficulties such as school drop-out and lower GPA, as well as reduced quality of life^8–10^ and substance abuse.^11^

Recreational screen time, which encompasses screen use unrelated to work or school, has averaged above 4 hours/day among undergraduates since the resolution of the COVID-19 pandemic.^12,13^ While screen time was already increasing,^14^ pandemic-related restrictions necessitated increases in screen time for entertainment and socialization. During this period, averages reached as high as 7 hours/day in undergraduates^15^ and have remained elevated compared to pre-pandemic levels.^16^ Higher daily screen time has been correlated with lower quality of life and well-being, heightened stress, and increased symptoms of depression, anxiety, and insomnia among young adults.^12,15,17–22^ Specifically, exceeding 3 hours/day of recreational screen time has been associated with increased depression and anxiety, and in some studies females appear more vulnerable.^19^ Other negative impacts include decreased physical activity/recreation, negative social comparison, reduced face-to-face connection, and increased risk of cyberbullying.^23–25^ While most studies have found negative associations between prolonged screen time and mental health, some report no impact or positive effects.^19,24,26^ Moderate screen use has been associated with better mental health, including higher self-esteem, resilience, and well-being.^24^ Longitudinal studies indicate that frequent social media use and leisure screen time can predict future mental health problems, but effects are generally small.^24^

While previous studies have largely focused on total recreational screen time and mental health it appears not all screen use is comparable.^24,26–28^ Unlike leisure screen time, social screen time involves active communication with others through video calling, texting, or messaging. While high levels of leisure screen time are largely seen as detrimental,^19,24,26,27^ moderate amounts of social screen time may be beneficial, allowing students to remain connected and feel supported.^29^ The nature and strength of associations between screen time and mental health appear to depend on the type of screen activity, duration of use, and individual factors such as gender and self-esteem.^24^

Few previous large-scale, longitudinal studies have examined recreational screen time and mental health in university students; few have considered differences by gender, and none to our knowledge have explored the moderating role of self-esteem. Examining these associations in first-year undergraduates is especially important, as they have less parental oversight and require more self-direction in screen time use. Further, they are often distanced from family and established friends and rely more on screens for academic commitments. For these reasons, university students may be particularly vulnerable to high levels of screen time use. Recreational screen time is often cited as a modifiable risk factor to address the risking rates of mental health concerns among university students, but its impact remains debated, requiring further investigation.

Utilizing longitudinal data from first-year undergraduates who began their studies after the peak of the COVID-19 pandemic (2021–2022 academic year), we examined associations between recreational screen time (leisure and social) at entry to university and mental health and well-being reported at the beginning and completion of the academic year, considering differences by gender. We also explored whether self-esteem influenced the associations between screen time and anxiety and depression. As a key psychological resource, self-esteem may affect recreational screen use and its impact on mental health,^30^ thereby guiding interventions targeting at-risk students.

## Methods

### Data Source

Study data were from the biannual U-Flourish Student Well-Being Survey study, the protocol of which is previously published.^31^ Briefly, since 2018, incoming first year students to Queen's University, Canada have been invited to complete an online survey in mid-September and again in mid-March, and biannually each year thereafter. The baseline survey collects data on students’ sociodemographic characteristics, daily behaviours, and mental and physical health. The U-Flourish student-led engagement team deploy a multifaceted strategy (e.g., booths on campus and at events, classroom talks, posters, and social media posts) to increase student participation.

This study included first-year students who completed a baseline survey in Fall 2021, after classes resumed in-person post COVID-19 pandemic, and some restrictions remained (e.g., mandatory masking and self-screening). The baseline survey, completed 2–3 weeks into the term, when students had settled into a routine, included supplemental items on daily social and leisure screen time. The fall baseline survey had a 34% response rate, and 41% of those respondents completed the spring follow-up. After reading a Letter of Information, students provided informed consent electronically and were given access to the survey hosted on Qualtrics. The U-Flourish Survey was reviewed for ethical compliance by the Queen's University Research Ethics Board (HSREB PSIY-609-18).

### Study Variables

#### Exposure to Recreational Screen Time at School Entry

Leisure screen time was defined as time spent passively watching television or videos, or using an application or game, while social screen time was defined as time engaged in actively communicating with others through screens (e.g., FaceTime, text messaging), unrelated to academic studies. Average daily hours of each type of screen activity were reported using the following options: ≤3 hours, 4–6, 7–9, 10–12, and 13 +  hours/day. Due to small cell sizes, the upper categories were collapsed into 10 +  hours/day for leisure screen time and 7 +  hours/day for social screen time.

#### Mental Health and Well-Being Outcomes at School Entry and Completion of the Academic Year

Symptoms of anxiety and depression over the past two weeks were measured using the 7-item Generalized Anxiety Disorder scale (GAD-7)^32^ and 9-item Patient Health Questionnaire (PHQ-9),^33^ respectively. On both scales a cut-off score of ≥10 indicates clinically significant symptoms.^32,33^ The eight-item Sleep Condition Indicator (SCI-8) measures past-month sleep quality, with scores of ≤16 indicating probable insomnia.^33^ Well-being was assessed using the 7-item Warwick-Edinburgh Mental Wellbeing Scale (SWEMWBS), with a cut-off of ≤19 used to indicate low well-being.^34^ Finally, the five-item SCOFF questionnaire was used to identify disordered eating behaviour.^35^ Bulimia nervosa and anorexia symptoms are assessed with ≥2 “Yes” responses indicating disordered eating.

#### Demographic Characteristics

Age in years was self-reported at school entry. Gender was self-identified by selecting from the following: male, female, non-binary, prefer not to say, as well as an option to self-describe their gender identity. International student status was self-reported. Participants reported their ethnicity based on a standard set of options which were collapsed into the following categories: White, Asian, Black, Other, and Multiple.^36^ Program of study was obtained by linking to the University administrative database.

#### Self-Esteem at School Entry (Effect Modifier)

Self-esteem was measured using the 10-item Rosenberg Self-Esteem Scale,^37^ and categorized using approximate tertiles into low (0–14), moderate (15–19), or high (20–30).

### Statistical Analysis

Analyses were conducted using SAS Version 9.4 (SAS Institute Inc., Cary, NC, USA). The sample was limited to students with complete data on age, gender, the screen time measures, and at least one mental health outcome, resulting in 1,562 of 1,943 students (80%) being retained for analysis. Analyses examining mental health outcomes at the end of the year included 678 students (43%). Recreational screen time and mental health concerns reported at baseline were not significantly different in those lost to follow-up compared to those who completed it (ꭓ^2^ *P* *>* 0.05).

Descriptive statistics were used to summarize the sample, average daily screen time, and the mental health outcomes. Spearman's correlation coefficient was used to assess the relationship between leisure and social screen time.^38^ Multivariable log-binomial regression was employed to examine the relative risk (RR) of screening positive for a mental health concern based on students’ screen time, adjusting for age and gender (if not stratified by gender). The first series of log-binomial regression models examined associations between screen time and mental health outcomes reported at school entry. Potential effect modification by gender (male vs. female) was tested using interaction terms. Where significant gender differences were indicated (*P* *<* 0.15), the stratified results were presented.

A second series of log-binomial regression models examined associations between screen time at school entry and mental health outcomes at year-end, adjusting for baseline outcome status to assess whether screen time was associated with emergent mental health problems. The upper categories of leisure screen time were further collapsed into 7 +  hours/day for these analyses due to small cell sizes. The regression analyses had 80% power (two-sided α=0.05) to detect RRs of 1.16–1.60 overall, with gender-stratified minimum detectable RRs of 1.68–2.06 (males) and 1.27–1.57 (females) for cross-sectional analyses, and 2.00–2.83 (males) and 1.34–1.71 (females) for the fully adjusted longitudinal models.

Effect modification by self-esteem of the associations between screen time and screening positive for anxiety and depression at university entry was explored by stratifying the models by levels of self-esteem.

## Results

### Description of the Sample

Most participants (n = 1,562) were 18 years old at school entry, identified as female, and either White (62%) or Asian (20%) ethnicity (Table 1). At university entry, the most common mental health screen-positives were for anxiety (45%) and depression (42%), followed by disordered eating (31%), probable insomnia (30%), and low well-being (28%) (Supplemental Table 1). Females more commonly screened positive than males (*ꭓ*^2^ *P* *<* 0.001), with the greatest differences observed for disordered eating (36% vs. 15%, *ꭓ*^2^ *P* *<* 0.001) and anxiety (50% vs. 29%, *ꭓ*^2^ *P* *<* 0.001). At school entry, average self-esteem was 17.1 out of 30 (SD = 5.7).

**Table 1.** Description of the Study Sample (n = 1,562).

	n	(%)
Age at baseline (Fall 2021)		
≤17	332	(20.6)
18	1036	(66.3)
19	86	(5.5)
≥20	118	(7.6)
Gender		
Female	1135	(72.7)
Male	394	(25.2)
Non-binary	28	(1.8)
Prefer not to say	5	(0.3)
Ethnicity		
White	967	(62.0)
Asian	309	(19.8)
Black	27	(1.7)
Other	76	(4.9)
Multiple	182	(11.7)
International student status		
Domestic (Canadian)	1480	(94.9)
International	79	(5.1)
Program of study		
Arts, Humanities, & Social sciences	550	(35.2)
Life & Physical sciences	402	(25.8)
Engineering & Applied sciences	168	(10.8)
Professional schools (Nursing, Med, Law)	162	(10.4)
Health sciences	129	(8.3)
Business	108	(6.9)
Computing	42	(2.7)
Self-esteem (0–30), Mean(SD)	17.1	(5.7)

*Note.* <1% missing data by variable.

### Recreational Screen Time at University Entry

**Figure 1.**
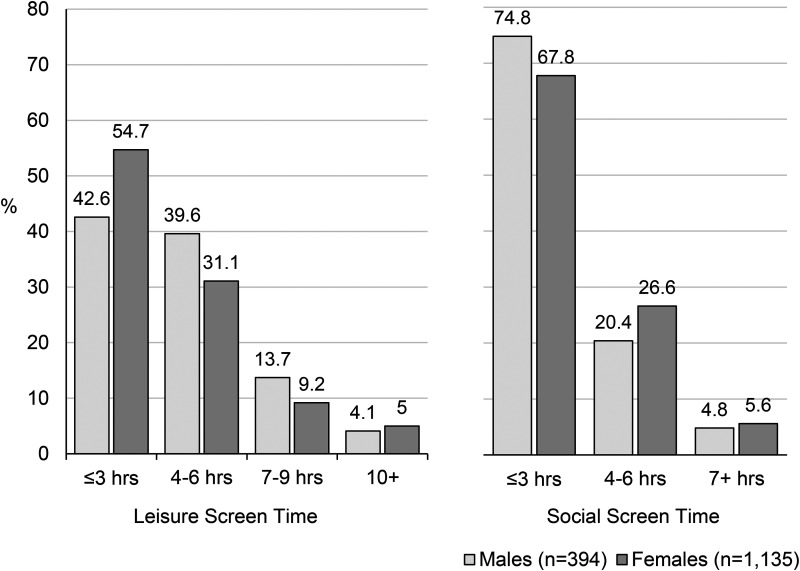
Average daily hours of leisure and social screen time reported by first-year undergraduates at school entry, by gender.

Figure 1 illustrates leisure and social screen time reported at university entry, stratified by gender. For leisure screen time, a higher percentage of males than females reported averaging 4 +  hours/day (57.4% vs. 45.3%, *ꭓ*^2^ *P* < 0.001), while for social screen time, a greater percentage of females reported 4 +  hours/day (32.2% vs. 25.2%, *ꭓ*^2^ *P* = 0.01). In total, 15.5% and 5.6% of students reported averaging 7 +  hours/day of leisure and social screen time, respectively. There was a weak correlation between leisure and social screen time (Spearman's rho = 0.22; 0.18 in males, 0.23 in females).

### Associations Between Screen Time at University Entry and Mental Health at Entry and Completion of First Year

At school entry, longer duration of daily screen time was associated with a greater likelihood of screening positive for common mental health problems, with stronger effects observed for leisure compared to social screen time (Table 2). Students reporting 7–9 and 10 +  hours/day of leisure screen time were 20–45% and 39–68% more likely to screen positive for anxiety, depression, insomnia, or low well-being than those averaging ≤3 hours/day (Table 2).

**Table 2.** Multivariable Log-Binomial Regression Results Examining Associations Between Average Daily Recreational Screen Time (Leisure and Social) at Entry to University and Screening Positive for Common Mental Health Problems at School Entry (Fall 2021) and the End of the Academic Year (Spring 2022).

		Anxiety (GAD-7 ≥ 10)			Depression (PHQ-9 ≥ 10)			Insomnia (SCI-8 ≤ 16)			Low Well-Being (WEMWBS-7 ≤ 19)		
		*Mental Health Concerns at School Entry*											
Leisure	n*	%Yes	RR	(95% CI)	%Yes	RR	(95% CI)	%Yes	RR	(95% CI)	%Yes	RR	(95% CI)
≤3 hours	800	42.3	1.00	ref	36.2	1.00	ref	27.0	1.00	ref	25.1	1.00	ref
4–6 hours	517	44.8	1.06	(0.94–1.20)	45.5	**1**.**24**	**(1.09–1.40)**	32.4	**1**.**22**	**(1.03–1.44)**	27.7	1.12	(0.94–1.35)
7–9 hours	164	50.6	**1**.**20**	**(1.01–1.43)**	54.3	**1**.**39**	**(1.18–1.63)**	32.9	1.22	(0.95–1.57)	37.2	**1**.**45**	**(1.14–1.84)**
10+ hours	77	62.3	**1**.**39**	**(1.14–1.70)**	58.4	**1**.**47**	**(1.18–1.82)**	46.8	**1**.**55**	**(1.17–2.05)**	41.6	**1**.**68**	**(1.68–2.26)**
Social													
≤3 hours	1081	41.6	1.00	ref	38.7	1.00	ref	28.5	1.00	ref	26.3	1.00	ref
4–6 hours	391	53.0	**1**.**15**	**(1.02–1.29)**	49.9	**1**.**14**	**(1.01–1.28)**	33.3	1.08	(0.91–1.29)	32.3	1.09	(0.92–1.30)
7 + hours	87	50.6	1.04	(0.83–1.31)	51.7	1.12	(0.90–1.40)	40.2	1.19	(0.89–1.58)	31.0	0.96	(0.69–1.35)
Model 1	*Mental Health Concerns at the End of the Academic Year*												
Leisure	n**	%Yes	RR	(95% CI)	%Yes	RR	(95% CI)	%Yes	RR	(95% CI)	%Yes	RR	(95% CI)
≤3 hours	362	45.1	1.00	ref	42.9	1.00	ref	32.6	1.00	ref	25.7	1.00	ref
4–6 hours	203	48.2	1.07	(0.89–1.29)	41.8	0.97	(0.79–1.19)	34.1	1.02	(0.79–1.33)	30.1	1.12	(0.72–1.41)
7 + hours	113	44.0	0.94	(0.73–1.21)	58.1	**1**.**27**	**(1.02–1.60)**	41.9	1.20	(0.88–1.63)	39.8	**1**.**49**	**(1.09–2.19)**
Social													
≤3 hours	478	45.1	1.00	ref	42.6	1.00	ref	33.5	1.00	ref	27.8	1.00	ref
4–6 hours	162	46.5	1.01	(0.83–1.23)	49.7	1.11	(0.91–1.35)	33.9	0.94	(0.72–1.24)	32.1	1.01	(0.71–1.35)
7 + hours	38	52.8	1.11	(0.80–1.56)	55.6	1.10	(0.79–1.52)	50.0	1.39	(0.93–2.06)	36.8	1.16	(0.73–2.05)
Model 2													
Leisure													
≤3 hours	362	45.1	1.00	ref	42.9	1.00	ref	32.5	1.00	ref	25.7	1.00	ref
4–6 hours	203	48.2	1.06	(0.92–1.21)	41.8	0.92	(0.79–1.08)	34.3	0.98	(0.86–1.12)	30.1	1.06	(0.86–1.30)
7 + hours	113	44.0	**0**.**80**	**(0.65–0.99)**	58.1	0.97	(0.81–1.18)	41.9	1.02	(0.86–1.20)	39.8	1.15	(0.91–1.45)
Social													
≤3 hours	478	45.1	1.00	ref	42.6	1.00	ref	33.5	1.00	ref	27.8	1.00	ref
4–6 hours	162	46.5	0.99	(0.85–1.15)	49.7	1.07	(0.91–1.26)	33.9	0.98	(0.85–1.13)	32.1	1.01	(0.82–1.23)
7 + hours	38	52.8	1.10	(0.83–1.46)	55.6	1.04	(0.82–1.33)	50.0	1.05	(0.86–1.28)	36.8	1.15	(0.81–1.63)

*Note.* (1) * < 0.4% missing data by outcome, (2) ** ≤ 20% missing data by outcome, (3) All models adjusted for age and gender, (4) Model 2 adjusted for age, gender, and baseline mental health symptoms, (5) statistically significant findings (*P* < 0.05) are bolded.

Associations between screen time and mental health outcomes were comparable between male and female students, except for disordered eating (Table 3). At school entry longer duration of leisure screen time was significantly associated with an increased risk of screening positive for disordered eating in females, but not males. However, 7 +  hours of daily leisure screen time increased the risk of screening positive for disordered eating at the end of the academic year in both males and females. While 4 +  hours/day of social screen time was associated with an increased risk of disordered eating at school entry, the effect was larger and statistically significant only in males (Table 3).

**Table 3.** Multivariable Log-Binomial Regression Results Examining Associations Between Screen Time Reported at School Entry and Screening Positive for Disordered Eating (SCOFF ≥2) at the Beginning and End of the Academic Year, Stratified by Gender.

	Beginning of the Academic Year				End of the Academic Year					
	Females				Females					
Leisure	n	%Yes	RR	(95% CI)	n	%Yes	RR	(95% CI)	*RR	(95% CI)
≤3 hours	620	31.9	1.00	ref	268	25.0	1.00	Ref	1.00	ref
4–6 hours	351	38.2	1.17	(0.98–1.40)	128	26.6	1.10	(0.78–1.56)	1.03	(0.77–1.38)
7–9/7 + hours	104	45.2	**1**.**30**	**(1.01–1.67)**	62	45.2	**1**.**66**	**(1.13–2.44)**	1.30	(0.94–1.80)
10+ hours	57	54.4	**1**.**55**	**(1.16–2.06)**						
Social										
≤3 hours	768	33.5	1.00	ref	322	27.3	1.00	Ref	1.00	ref
4–6 hours	301	40.5	1.13	(0.95–1.35)	109	23.9	0.77	(0.52–1.13)	0.83	(0.60–1.15)
7 + hours	64	48.4	1.22	(0.91–1.64)	27	55.6	**1**.**62**	**(1.05–2.50)**	1.13	(0.78–1.62)

Leisure	Males				Males					
	n	%Yes	RR	(95% CI)	n	%Yes	RR	(95% CI)	*RR	(95% CI)
≤3 hours	167	15.0	1.00	ref	62	11.3	1.00	ref	1.00	ref
4–6 hours	156	14.1	0.90	(0.53–1.52)	57	17.5	1.51	(0.61–3.70)	1.38	(0.66–2.88)
7 +	70	14.3	0.87	(0.44–1.74)	35	31.4	**3**.**04**	**(1.29–7.19)**	1.67	(0.87–3.20)
Social										
≤3 hours	294	12.6	1.00	ref	114	18.4	1.00	ref	1.00	ref
4 + hours	99	20.2	**1**.**70**	**(1.03–2.81)**	40	17.5	0.84	(0.39–1.83)	0.79	(0.45–1.38)

*Note.* (1) Models adjusted for age, and the other screen time measure, (2) * Models adjusted for age, the other screen time measure, and baseline screen positive status for disordered eating, (3) statistically significant findings (*P* < 0.05) are bolded.

Associations between screen time at school entry and mental health at the end of the year were generally consistent with the cross-sectional analysis, but smaller in magnitude (Table 2). However, only the associations between leisure screen time (≥7 hours/day) and increased risk of screening positive for depression and low well-being remained statistically significant; the association with anxiety was no longer evident (Tables 2 and 3). After adjustment for baseline symptoms, the associations were no longer statistically significant.

### Self-Esteem as a Moderator of the Relationship Between Screen Time and Anxiety and Depression at University Entry

Students with low self-esteem were significantly more likely to screen positive for anxiety (72.5%) and depression (77.6%) at school entry than those with moderate (43.9% and 37.5%) and high (18.0% and 11.8%) self-esteem. However, the cross-sectional associations between longer duration of leisure screen time and clinically significant levels of anxiety and depressive symptoms were only observed in the high self-esteem group (Table 4). High self-esteem students who reported averaging 4–6 and 7 +  hours/day of leisure screen time were 73% and 139% more likely to screen positive for anxiety and 118% and 172% more likely to screen positive for depression than those reporting ≤3 hours/day, respectively.

**Table 4.** Multivariable Log-Binomial Regression Results Exploring Associations Between Average Daily Recreational Screen Time (Leisure and Social) and Screening Positive for Anxiety and Depression at University Entry (Fall 2021), Stratified by Level of Self-Esteem.

	Low Self-Esteem				Moderate Self-Esteem				High Self-Esteem			
	Outcome: Anxiety Screen Positive (GAD-7 ≥ 10)											
Leisure	n	%Yes	RR	(95% CI)	n	%Yes	RR	(95% CI)	n	%Yes	RR	(95% CI)
≤3 hours	207	79.2	1.00	ref	312	43.3	1.00	ref	280	13.6	1.00	ref
4–6 hours	190	64.7	0.83	(0.65–1.05)	171	44.4	1.01	(0.76–1.34)	155	20.7	**1**.**73**	**(1.07–2.78)**
7 + hours	104	73.1	0.93	(0.70–1.23)	82	45.1	1.03	(0.70–1.52)	55	32.7	**2**.**39**	**(1.29–4.40)**
Social												
≤3 hours	334	71.6	1.00	ref	383	39.7	1.00	ref	363	16.0	1.00	ref
4–6 hours	138	73.9	1.02	(0.81–1.30)	146	55.5	**1**.**35**	**(1.03–1.77)**	105	21.9	1.07	(0.64–1.78)
7 + hours	28	75.9	1.05	(0.67–1.64)	36	41.7	1.03	(0.58–1.81)	22	31.8	1.52	(0.68–3.43)

Leisure	Outcome: Depression Screen Positive (PHQ-9 ≥ 10)												
	n	%Yes	RR	(95% CI)	n	%Yes	RR	(95% CI)	n	%Yes	RR	(95% CI)
≤3 hours	206	78.2	1.00	ref	312	33.7	1.00	ref	280	7.9	1.00	ref
4–6 hours	189	75.1	0.98	(0.78–1.23)	172	40.1	1.15	(0.84–1.56)	156	15.4	**2**.**18**	**(1.21–3.92)**
7 + hours	104	80.8	1.03	(0.78–1.35)	82	46.3	1.34	(0.89–2.00)	55	21.8	**2**.**72**	**(1.28–5.76)**
Social												
≤3 hours	332	75.6	1.00	ref	383	33.9	1.00	ref	363	9.9	1.00	ref
4–6 hours	138	80.4	1.05	(0.83–1.31)	147	45.6	1.29	(0.95–1.74)	106	16.0	1.18	(0.64–2.16)
7 + hours	29	86.2	1.10	(0.72–1.67)	36	41.7	1.08	(0.61–1.92)	22	22.7	1.74	(0.66–4.60)

*Note.* (1) < 0.4% missing data by outcome, (2) All models adjusted for age, gender, and the other screen time measure, (3) statistically significant findings (*P* < 0.05) are bolded.

## Discussion

This study examined the relationship between recreational screen time (leisure and social) and screening positive for common mental health concerns and low well-being in a large cohort of first-year undergraduates attending a major Canadian university. There was evidence of an association between averaging >3 hours/day of leisure screen time and screening positive for depression, anxiety, low well-being, and probable insomnia at university entry. Over half the students reported leisure screen time (4 +  hours/day) associated with increased risk of significant mental health concerns. Similarly, there was evidence of an association between 7 + hours/day of leisure screen time and screening positive for depression and low well-being at the end of the academic year; however, the associations became non-significant after adjustment for baseline symptoms. Finally, the associations between increased daily leisure screen time (4 +  hours) and screening positive for anxiety and depression at school entry were strongest in students reporting higher self-esteem.

Our main findings align with the extant literature. That is, while screen time has been associated with varying effects depending on individual differences, and the nature (passive vs. interactive; leisure vs. social), frequency, intensity, and duration of use, high levels of leisure screen time have consistently been linked with poor mental health.^19,26,39^ The positive relationship between duration of leisure screen time and increased risk of mental health problems identified is consistent with prior research on anxiety, depression, and insomnia.^40–44^ In this study, leisure screen time was associated with concurrent mental health status, but not with declines over the year, suggesting screen time may have a greater impact on current symptoms than the development of future problems. The observed cross-sectional association may be partly explained by reverse causality or shared underlying factors. Students experiencing poor mental health may use screens for emotional regulation, coping, or social connection, or because they lack the energy for non-screen activities.^45,46^ However, previous studies found limited evidence of a reverse association between psychopathology and subsequent screen time.^26^ Our finding of minimal long-term effects of screen time on mental health aligns with previous studies showing inconsistent and largely attenuated longitudinal associations.^26^

With the exception of disordered eating, screen time was positively correlated with mental health problems independent of gender, which differs from previous studies reporting more negative effects of high levels of screen time in females.^19,24,26^ However, these studies focused specifically on social media-based screen use.

The literature reports mixed findings regarding the effects of different types of screen time on mental health. While >3 hours/day of passive leisure screen time has consistently been linked to mood and anxiety disorders,^19,27^ some studies report no association or even positive effects on mental health.^27,47^ Similar to prior research, we found greater negative effects associated with leisure compared to active social screen time.^19,24,26,27^ Social media use has been negatively correlated with mental health, but few studies have examined the effects of time spent using screens specifically for interacting socially. In this study, social media-based screen time could have been captured as social (actively posting or messaging) or leisure (passively scrolling). Although not directly measured, our findings suggest passive social media use may have a greater negative mental health impact than active communication.

The passive use hypothesis posits that passive leisure screen time is associated with increased loneliness and negative affect, and that it increases upward social comparison more than active use.^48^ In contrast, using screens to connect with others socially has been associated with increased social connectedness, subjective well-being, and participation in other health-promoting activities including physical recreation.^29,49^ Although online interactions pose barriers to understanding tone, mood and body language, and decrease the overall level of social connectedness compared to in-person interactions,^50^ they may still alleviate feelings of loneliness.^51^

Interestingly, leisure screen time was significantly associated with disordered eating in females, but not males. This aligns with prior findings and highlights the importance of considering the type of screen use young males and females engage in. Leisure screen time, as defined in this study, encompasses different activities such as watching television or videos, playing video games, or scrolling on social media. Males and females may differ in how they use screens, and what they are consuming. Male respondents may have spent more time playing video games, while females may have spent more time scrolling on social media. Among 13–15-year-olds, girls were found to average 3.28 hours/day on social media, compared to 2.05 hours for boys.^28^ In contrast, boys spent significantly more time on video games (3.25 hours/day vs. 1.17). Importantly, social media use was more strongly associated with negative mental health outcomes than gaming.^28^ Specifically, exposure to appearance-focused social media has been linked to body image concerns and thin-ideal internalization among young women.^52^ Studies should further investigate how gendered patterns of screen use may influence disordered eating.^53^

### Self-Esteem at School Entry (Effect Modifier)

We found evidence that leisure screen time over 3 hours/day was most strongly associated with screening positive for anxiety and depression in students reporting higher self-esteem. This is a surprising finding, as higher self-esteem has been associated with more positive or protective effects on mental health and loneliness related to social media use,^54,55^ but there is little evidence to support a negative moderating effect on screen time and mental health. Previous research has also shown high self-esteem may only be protective up to a point, with effects diminishing at higher levels of screen time.^56^ Further research is needed to elucidate the mechanisms underlying this finding given the cross-sectional nature of the data and reliance on self-report.

### Implications and Future Directions

Recreational screen time greater than 3 hours/day was reported by over 50% of students in this study and represents a potentially modifiable target for health promotion and prevention initiatives in university students. While guidelines on screen time duration for young adults are unclear, and what constitutes “excessive” use remains debated, some recommend limiting recreational screen time to 3 hours daily.^57^ This aligns with our finding that over 3 hours/day of leisure screen time is associated with increased risk of common mental health concerns. Previous studies have explored ways to limit screen time by self-nudging apps that monitor and remind users that they have extended beyond their set time limit.^58^ Users of the app reported increased satisfaction with their use of screens and reduced time watching videos compared to controls.^59^ Other strategies include choosing screen activities that are more cognitively engaging and avoiding screens before bedtime.

Future research is needed to understand mechanisms by which screen time affects mental health or vice versa, such as impacts on sleep quality/duration, emotional regulation, loneliness, and physical activity. The specific content being consumed may be increasing depressive and anxiety symptoms in this population, and/or screen time may take time away from positive mental health practices, or the actual light emissions could increase symptoms for example.^26^ Understanding these mechanisms can help tailor university health promotion efforts to student behavioural patterns. Consideration of specific types of screen activities and applying objective measures^60^ can be used to further increase understanding of these associations.

### Strengths and Limitations

Strengths of our study include the use of a large broadly representative sample of first-year students,^61^ with prospective follow-up data. Validated screening measures for assessing mental health problems and self-esteem were used, enhancing the reliability of the findings. Limitations of this study include a bias toward over-sampling female students and loss to follow-up, which may affect generalizability. The screen time measures lacked detail and are susceptible to recall bias and measurement error. Notably, the lowest category of daily screen time was ≤3 hours, which may have masked variability in risk. Additionally, the use of a single time-point measure of screen use may not have captured more habitual use. While we were able to examine leisure versus active social screen time, we did not have detailed or objective measures of either, and we did not specifically examine social media use. Furthermore, students may have had difficulty separating leisure and social screen time, which may not be mutually exclusive. Consequently, our ability to identify more nuanced associations between screen time and mental health was limited. Lastly, this study had limited power to detect some associations, particularly at higher levels of screen time and within gender-stratified analyses.

## Conclusion

This study found evidence that leisure screen time of 4 +  hours/day at entry to university is common and negatively associated with mental health among first-year undergraduates. Future research is needed on the mechanisms underlying the negative impact of screen time on university students’ mental health, and those most at risk. However, there appears to be sufficient evidence and rationale to include guidance on healthy and responsible recreational screen time for incoming university students as part of mental health prevention and health promotion initiatives.

## Supplemental Material

sj-docx-1-cpa-10.1177_07067437261428821 - Supplemental material for Recreational Screen Time at University Entry and Mental Health and Well-Being Over First Year: U-Flourish Student Well-Being Research Supplemental material, sj-docx-1-cpa-10.1177_07067437261428821 for Recreational Screen Time at University Entry and Mental Health and Well-Being Over First Year: U-Flourish Student Well-Being Research by Simran Brar, MSc, Nathan King, PhD, Anna Park, BHSc, Kristen Kyone, BHSc, Emily Dephoure, BHSc, Daniel Rivera, MSc, Adeleine Lyon, MSc and Anne Duffy, MD, MSc, FRCPC in The Canadian Journal of Psychiatry

## References

[1] Ding F Yu B . First year university students’ perception of autonomy: an individualistic approach. J Furth High Educ. 2022;46(2):211–224.

[2] Duffy A Saunders KEA Malhi GS , et al. Mental health care for university students: a way forward? Lancet Psychiatry. 2019;6(11):885–887. 31324561 10.1016/S2215-0366(19)30275-5

[3] King N Pickett W McNevin SH , et al. Mental health need of students at entry to university: baseline findings from the U-Flourish Student Well-being and Academic Success Study. Early Interv Psychiatry. 2021;15(2):286–295. 32048460 10.1111/eip.12939

[4] Li W Zhao Z Chen D , et al. Prevalence and associated factors of depression and anxiety symptoms among college students: a systematic review and meta-analysis. J Child Psychol Psychiatry. 2022;63(11):1222–1230. 35297041 10.1111/jcpp.13606

[5] Appleby JA King N Saunders KE , et al. Impact of the COVID-19 pandemic on the experience and mental health of university students studying in Canada and the UK: a cross-sectional study. BMJ Open. 2022;12(1):e050187. 10.1136/bmjopen-2021-050187 PMC8787844 35074809

[6] Fruehwirth JC Biswas S Perreira KM . The Covid-19 pandemic and mental health of first-year college students: examining the effect of Covid-19 stressors using longitudinal data. PLOS ONE. 2021;16(3):e0247999. 10.1371/journal.pone.0247999 PMC7935268 33667243

[7] King N Pickett W Rivera D , et al. The impact of the COVID-19 pandemic on the mental health of first-year undergraduate students studying at a Major Canadian University: a successive cohort study. Can J Psychiatry Rev Can Psychiatr. 2023;68(7):499–509. 10.1177/07067437221094549 PMC9096012 35450455

[8] Bruffaerts R Mortier P Kiekens G , et al. Mental health problems in college freshmen: prevalence and academic functioning. J Affect Disord. 2018;225:97–103. 28802728 10.1016/j.jad.2017.07.044 PMC5846318

[9] Duffy A Keown-Stoneman C Goodday S , et al. Predictors of mental health and academic outcomes in first-year university students: identifying prevention and early-intervention targets. BJPsych Open. 2020;6(3):e46. 10.1192/bjo.2020.24 PMC7331085 32381150

[10] Fernandes MdS Mendonça CR da Silva TMV , et al. Relationship between depression and quality of life among students: a systematic review and meta-analysis. Sci Rep. 2023;13(1):6715. 37185375 10.1038/s41598-023-33584-3 PMC10126541

[11] Walters KS Bulmer SM Troiano PF , et al. Substance use, anxiety, and depressive symptoms among college students. J Child Adolesc Subst Abuse. 2018;27(2):103–111.

[12] Lavados-Romo P Andrade-Mayorga O Morales G , et al. Association of screen time and physical activity with health-related quality of life in college students. J Am Coll Health. 2023;71(5):1504–1509. 34242535 10.1080/07448481.2021.1942006

[13] Pellerine LP Bray NW Fowles JR , et al. Increased recreational screen time and time to fall asleep are associated with worse academic performance in Canadian undergraduates. Int J Health Promot Educ. 2023. Advance online publication. https://doi.org/10.1080/14635240.2023.2248091.

[14] Government of Canada SC. Sociodemographic differences in recreational screen time before and during the COVID-19 pandemic in Canada. https://www150.statcan.gc.ca/n1/pub/82-003-x/2024005/article/00001-eng.htm (2024, accessed October 4, 2024).

[15] Deyo A Wallace J Kidwell KM . Screen time and mental health in college students: time in nature as a protective factor. J Am Coll Health. 2024;72(8):3025–3032. 36796079 10.1080/07448481.2022.2151843

[16] Government of Canada SC. Physical activity and screen time: Pandemic effects, and other key numbers. https://www.statcan.gc.ca/o1/en/plus/4989-physical-activity-and-screen-time-pandemic-effects-and-other-key-numbers (2023, accessed October 4, 2024).

[17] Carter B Rees P Hale L , et al. Association between portable screen-based Media device access or use and sleep outcomes: a systematic review and meta-analysis. JAMA Pediatr. 2016;170(12):1202–1208. 27802500 10.1001/jamapediatrics.2016.2341 PMC5380441

[18] Gao XL Zhang JH Yang Y , et al. Sedentary behavior, screen time and mental health of college students: a meta-analysis. Zhonghua Liu Xing Bing Xue Za Zhi Zhonghua Liuxingbingxue Zazhi. 2023;44(3):477–485. 36942345 10.3760/cma.j.cn112338-20220728-00669

[19] Hilty DM Stubbe D McKean AJ , et al. A scoping review of social media in child, adolescents and young adults: research findings in depression, anxiety and other clinical challenges. BJPsych Open. 2023;9(5):e152. 10.1192/bjo.2023.523 PMC10594088 37563766

[20] Hjetland GJ Skogen JC Hysing M , et al. The association between self-reported screen time, social media addiction, and sleep among Norwegian University students. Front Public Health. 2021;9:794307. 34976935 10.3389/fpubh.2021.794307 PMC8716598

[21] Rosenthal SR Zhou J Booth ST . Association between mobile phone screen time and depressive symptoms among college students: a threshold effect. Hum Behav Emerg Technol. 2021;3(3):432–440.

[22] Zahedi S Jaffer R Iyer A . A systematic review of screen-time literature to inform educational policy and practice during COVID-19. Int J Educ Res Open. 2021;2:100094. 35059672 10.1016/j.ijedro.2021.100094 PMC8592820

[23] Barlett ND Gentile DA Barlett CP , et al. Sleep as a mediator of screen time effects on US children’s health outcomes: a prospective study. J Child Media. 2012;6(1):37–50.

[24] Santos RMS Mendes CG Sen Bressani GY , et al. The associations between screen time and mental health in adolescents: a systematic review. BMC Psychol. 2023;11(1):127. 37081557 10.1186/s40359-023-01166-7 PMC10117262

[25] Mantey DS Yockey RA Springer AE . Digital screen time and suicidality during high school: how important is cyberbullying? A mediation analysis using the youth risk behavioral surveillance survey, 2011–2019. Prev Med. 2023;166:107330. 36334685 10.1016/j.ypmed.2022.107330

[26] Tang S Werner-Seidler A Torok M , et al. The relationship between screen time and mental health in young people: a systematic review of longitudinal studies. Clin Psychol Rev. 2021;86:102021. 33798997 10.1016/j.cpr.2021.102021

[27] Kim S Favotto L Halladay J , et al. Differential associations between passive and active forms of screen time and adolescent mood and anxiety disorders. Soc Psychiatry Psychiatr Epidemiol. 2020;55(11):1469–1478. 32055896 10.1007/s00127-020-01833-9

[28] Twenge JM Farley E . Not all screen time is created equal: associations with mental health vary by activity and gender. Soc Psychiatry Psychiatr Epidemiol. 2021;56(2):207–217. 32743778 10.1007/s00127-020-01906-9

[29] Liang N Grayson SJ Kussman MA , et al. In-person and virtual social interactions improve well-being during the COVID-19 pandemic. Comput Hum Behav Rep. 2024;15:100455.

[30] Kim Y Lee M . Can self-esteem protect the subjective well-being of women in their 20s from the effects of social media use? The moderating role of self-esteem. Behav Sci. 2025;15(7):964. 40723749 10.3390/bs15070964 PMC12292396

[31] Goodday SM Rivera D Foran H , et al. U-Flourish university students well-being and academic success longitudinal study: a study protocol. BMJ Open. 2019;9(8):e029854. 10.1136/bmjopen-2019-029854 PMC6720248 31455708

[32] Spitzer RL Kroenke K Williams JBW , et al. A brief measure for assessing generalized anxiety disorder: the GAD-7. Arch Intern Med. 2006;166(10):1092–1097. 16717171 10.1001/archinte.166.10.1092

[33] Kroenke K Spitzer RL Williams JBW . The PHQ-9. J Gen Intern Med. 2001;16(9):606–613. 11556941 10.1046/j.1525-1497.2001.016009606.x PMC1495268

[34] Stewart-Brown. The Warwick-Edinburgh Mental Wellbeing Scale (WEMWBS). https://warwick.ac.uk/fac/sci/med/research/platform/wemwbs (2015, accessed April 8, 2025).

[35] Morgan JF Reid F Lacey JH . The SCOFF questionnaire: assessment of a new screening tool for eating disorders. Br Med J. 1999;319(7223):1467–1468. 10582927 10.1136/bmj.319.7223.1467 PMC28290

[36] Government of Canada SC. Ethnic or Cultural Origin Reference Guide, Census of Population, 2021. https://www12.statcan.gc.ca/census-recensement/2021/ref/98-500/008/98-500-x2021008-eng.cfm (2022, accessed July 3, 2025).

[37] Rosenberg M . Society and the Adolescent Self-Image. Princeton (NJ): Princeton University Press; 1965. https://www.jstor.org/stable/j.ctt183pjjh (accessed January 7, 2025).

[38] Akoglu H . User’s guide to correlation coefficients. Turk J Emerg Med. 2018;18(3):91–93. 30191186 10.1016/j.tjem.2018.08.001 PMC6107969

[39] Neophytou E Manwell LA Eikelboom R . Effects of excessive screen time on neurodevelopment, learning, memory, mental health, and neurodegeneration: a scoping review. Int J Ment Health Addict. 2021;19(3):724–744.

[40] Grøntved A Singhammer J Froberg K , et al. A prospective study of screen time in adolescence and depression symptoms in young adulthood. Prev Med. 2015;81:108–113. 26303369 10.1016/j.ypmed.2015.08.009

[41] Gunnell KE Flament MF Buchholz A , et al. Examining the bidirectional relationship between physical activity, screen time, and symptoms of anxiety and depression over time during adolescence. Prev Med. 2016;88:147–152. 27090920 10.1016/j.ypmed.2016.04.002

[42] Liu M Wu L Yao S . Dose–response association of screen time-based sedentary behaviour in children and adolescents and depression: a meta-analysis of observational studies. Br J Sports Med. 2016;50(20):1252–1258. 26552416 10.1136/bjsports-2015-095084 PMC4977203

[43] Maras D Flament MF Murray M , et al. Screen time is associated with depression and anxiety in Canadian youth. Prev Med. 2015;73:133–138. 25657166 10.1016/j.ypmed.2015.01.029

[44] Vézina-Im L-A Beaulieu D Turcotte S , et al. Association between recreational screen time and sleep quality among adolescents during the third wave of the COVID-19 pandemic in Canada. Int J Environ Res Public Health. 2022;19(15):9019. 35897389 10.3390/ijerph19159019 PMC9332431

[45] Deatherage S Servaty-Seib HL Aksoz I . Stress, coping, and internet use of college students. J Am Coll Health. 2014;62(1):40–46. 24313695 10.1080/07448481.2013.843536

[46] Pera A . The psychology of addictive smartphone behavior in young adults: problematic use, social anxiety, and depressive stress. Front Psychiatry. 2020;11:573473. 33101087 10.3389/fpsyt.2020.573473 PMC7522217

[47] Woods HC Scott H . #Sleepyteens: social media use in adolescence is associated with poor sleep quality, anxiety, depression and low self-esteem. J Adolesc. 2016;51:41–49. 27294324 10.1016/j.adolescence.2016.05.008

[48] Meier A Krause H-V . Does passive social media use harm well-being? J Media Psychol. 2023;35(3):169–180.

[49] Tay L Tan K Diener E , et al. Social relations, health behaviors, and health outcomes: a survey and synthesis. Appl Psychol Health Well-Being. 2013;5(1):28–78. 23281315 10.1111/aphw.12000

[50] Scott RA Stuart J Barber BL , et al. Social connections during physical isolation: How a shift to online interaction explains friendship satisfaction and social well-being. Cyberpsychol J Psychosoc Res Cyberspace. 2022;16(2):10.

[51] Ellis WE Dumas TM Forbes LM . Physically isolated but socially connected: psychological adjustment and stress among adolescents during the initial COVID-19 crisis. Can J Behav Sci Rev Can Sci Comport. 2020;52(3):177–187.

[52] Cohen R Newton-John T Slater A . The relationship between Facebook and Instagram appearance-focused activities and body image concerns in young women. Body Image. 2017;23:183–187. 29055773 10.1016/j.bodyim.2017.10.002

[53] Perloff RM . Social Media effects on young women’s body image concerns: theoretical perspectives and an agenda for research. Sex Roles. 2014;71(11):363–377.

[54] Lin S Liu D Niu G , et al. Active social network sites use and loneliness: the mediating role of social support and self-esteem. Curr Psychol. 2022;41(3):1279–1287.

[55] Liu W Yu J She X , et al. Chain mediating role of self-esteem and resilience in the association between screen time and depression symptoms among Chinese children and adolescents. Curr Psychol. 2024;43(43):33368–33381.

[56] Rosenthal SR Tobin AP . Self-esteem only goes so far: the moderating effect of social media screen time on self-esteem and depressive symptoms. Behav Inf Technol. 2023;42(15):2688–2695. 37994349 10.1080/0144929x.2022.2139759 PMC10662696

[57] Ross R Chaput J-P Giangregorio LM , et al. Canadian 24-Hour Movement guidelines for adults aged 18–64 years and adults aged 65 years or older: an integration of physical activity, sedentary behaviour, and sleep. Appl Physiol Nutr Metab. 2020;45(10 (Suppl. 2)):S57–S102. 10.1139/apnm-2020-0467 33054332

[58] Olson JA Sandra DA Chmoulevitch D , et al. A nudge-based intervention to reduce problematic smartphone use: randomised controlled trial. Int J Ment Health Addict. 2022;21:3842–3864. 10.1007/s11469-022-00826-w PMC9112639 35600564

[59] Grüning DJ Riedel F Lorenz-Spreen P . Directing smartphone use through the self-nudge app one sec. Proc Natl Acad Sci U S A. 2023;120(8):e2213114120. 10.1073/pnas.2213114120 PMC9974409 36795756

[60] Katapally TR Chu LM . Methodology to derive objective screen-state from smartphones: a SMART platform study. Int J Environ Res Public Health. 2019;16(13):2275. 31252617 10.3390/ijerph16132275 PMC6651165

[61] Queen’s University 2021-22 Enrolment Report.

