# Supplementary material for: Recreational Screen Time at University Entry and Mental Health and Well-Being Over First Year: U-Flourish Student Well-Being Research: Temps d’écran à des fins de divertissement au moment de l’entrée à l’université, santé mentale et bien-être au cours de la première année : programme de recherche U-Flourish sur le bien-être des étudiants
Source: Can J Psychiatry. 2026 Mar 6:07067437261428821. Online ahead of print. doi: 10.1177/07067437261428821 (PMC12965894; doi:10.1177/07067437261428821)
Supplement: sj-docx-1-cpa-10.1177_07067437261428821 - Supplemental material for Recreational Screen Time at University Entry and Mental Health and Well-Being Over First Year: U-Flourish Student Well-Being Research [file sj-docx-1-cpa-10.1177_07067437261428821.docx]

| **Supplementary Table 1**. Description of screen positives for common mental health problems at school entry and the end of the academic year in the full sample of first-year undergraduate students, stratified by gender | | | | | | | | | | | | | |
| --- | --- | --- | --- | --- | --- | --- | --- | --- | --- | --- | --- | --- | --- |
|  | **Full Sample** | | | | **Males** | | | | **Females** | | | | p* |
| ***School Entry:*** | nTot | nYes | %Yes | (95% CI) | nTot | nYes | %Yes | (95% CI) | nTot | nYes | %Yes | (95% CI) |  |
| Anxiety (GAD-7 ≥10) | 1557 | 700 | 45.0 | (42.5-47.4) | 392 | 114 | 29.1 | (24.6-33.6) | 1132 | 562 | 49.6 | (46.7-52.6) | <.001 |
| Depression (PHQ-9 ≥10) | 1557 | 658 | 42.3 | (39.8-44.7) | 392 | 117 | 29.8 | (25.3-34.4) | 1132 | 515 | 45.5 | (42.6-48.4) | <.001 |
| Insomnia (SCI-8 ≤16) | 1553 | 472 | 30.4 | (28.1-32.7) | 392 | 93 | 23.7 | (19.5-28.0) | 1128 | 363 | 32.2 | (29.5-34.9) | 0.002 |
| Disordered Eating (SCOFF ≥2) | 1558 | 479 | 30.7 | (28.5-33.0) | 393 | 57 | 14.5 | (11.0-18.0) | 1132 | 410 | 36.2 | (33.4-39.0) | <.001 |
| Low Well-being (WEMWBS-7 ≤19) | 1558 | 437 | 28.0 | (25.8-30.3) | 393 | 69 | 17.6 | (13.8-21.3) | 1132 | 350 | 30.9 | (28.2-33.6) | <.001 |
| ***End of the Academic Year:*** |  |  |  |  |  |  |  |  |  |  |  |  |  |
| Anxiety (GAD-7 ≥10) | 665 | 305 | 45.9 | (42.1-49.7) | 161 | 60 | 37.3 | (29.7-44.8) | 479 | 229 | 47.8 | (43.3-52.3) | 0.02 |
| Depression (PHQ-9 ≥10) | 642 | 289 | 45.0 | (41.2-48.9) | 154 | 64 | 41.6 | (33.7-49.4) | 464 | 210 | 45.3 | (40.7-49.8) | 0.42 |
| Insomnia (SCI-8 ≤16) | 544 | 188 | 34.6 | (30.6-38.6) | 128 | 42 | 32.8 | (24.6-41.1) | 394 | 133 | 33.8 | (29.1-38.4) | 0.84 |
| Disordered Eating (SCOFF ≥2) | 636 | 163 | 25.6 | (22.2-29.0) | 154 | 28 | 18.2 | (12.0-24.3) | 458 | 129 | 28.2 | (24.0-32.3) | 0.01 |
| Low Well-being (WEMWBS-7 ≤19) | 678 | 199 | 29.4 | (25.9-32.8) | 164 | 46 | 28.0 | (21.1-35.0) | 489 | 139 | 28.4 | (24.4-32.4) | 0.93 |
| *Note: *p-value from chi-square test comparing % reporting the outcome by gender (male vs. female)* | | | | | | | | | | | | | |
